# Impact of a randomized weight loss trial on breast tissue markers in breast cancer survivors

**DOI:** 10.1038/s41523-022-00396-z

**Published:** 2022-03-07

**Authors:** Christina M. Dieli-Conwright, Maura Harrigan, Brenda Cartmel, Anees Chagpar, Yalai Bai, Fang-yong Li, David L. Rimm, Lajos Pusztai, Lingeng Lu, Tara Sanft, Melinda L. Irwin

**Affiliations:** 1 Division of Population Sciences, Department of Medical Oncology, Dana-Farber Cancer Institute, Harvard Medical School, Boston, MA USA; 2 Yale School of Public Health, New Haven, CT USA; 3 Yale Cancer Center, New Haven, CT USA; 4 Yale School of Medicine, New Haven, CT USA; 5 Yale Center for Analytic Sciences, New Haven, CT USA

**Keywords:** Prognostic markers, Diagnostic markers

## Abstract

Few trials have examined the effect of lifestyle behavioral interventions on tissue markers in patients with cancer. The purpose of this study was to examine the feasibility and impact of a 6-month weight loss intervention on breast tissue and serum biomarkers in women with breast cancer. Fifty-one women who completed breast cancer treatment and had a BMI ≥ 25.0 kg/m^2^ were randomized to a weight loss intervention or usual care. Breast tissue biopsies, fasting blood draw and body composition were collected at baseline and 6 months, with between-group changes examined using analysis of covariance method. Baseline and post-intervention biopsies were conducted in 49 and 42 women, respectively, with pre- and post-epithelial tissue available from 25 tissue samples. Average 6-month weight loss was 6.7% for the weight loss group and 2.0% increase for the usual care group (*p* < 0.0001). At baseline, body fat and serum insulin levels were inversely associated with breast tissue insulin receptor levels and CD68 (*p* < 0.05). At 6 months, favorable changes were observed in serum leptin and adiponectin levels and tissue CD163 among women randomized to weight loss vs. adverse change in women randomized to usual care (*p* < 0.05). Breast tissue biopsies are feasible to collect in a clinical research setting among breast cancer survivors, with weight loss favorably impacting metabolic and inflammatory markers associated with breast cancer.

## Introduction

Obesity is a risk factor for 13 different cancers^1^, with mounting evidence that it may additionally play a role in mortality from breast cancer^2^. The American Cancer Society recommends cancer survivors achieve and maintain healthy body weight, avoid physical inactivity, participate in 150 min of aerobic exercise plus two weekly sessions of strength training, and adhere to a dietary pattern high in vegetables, fruits, and whole grains^3^. Unfortunately, over 65% of breast cancer survivors are overweight or obese, with fewer than 30% engaging in recommended levels of physical activity^4,5^.

Possible mechanisms through which obesity affects breast cancer risk and mortality include inflammatory, immune, metabolic, and hormonal pathways^6,7^. A growing number of studies have examined serum biomarkers such as adiponectin, insulin, C-reactive protein, IL-6, TNF-α, and particularly leptin as said biomarkers are associated with these mechanisms in exercise and/or weight loss trials in cancer survivors or those at risk of developing cancer^8–12^.

However, examining changes in tissue markers provides a more direct investigation of the impact of lifestyle interventions on tumor biology and allows for the assessment of underlying mechanistic pathways that cannot be examined from blood-based biomarkers alone. Preliminary evidence suggests that weight loss and/or exercise interventions may impact tumor biology in both breast cancer patients^9,10^ and in women at increased breast cancer risk due to high body mass index (Table 1)^8^. Said tissue biomarkers include tumor biomarkers (e.g., Ki67), inflammatory macrophage biomarkers of the M1 and M2 phenotype (e.g., CD163 and CD68), and biomarkers related to insulin (e.g., insulin receptor) strongly associated with recurrent breast cancer^13^.

**Table 1:** Tissue changes induced by nutrition and exercise interventions.

	Population	Tissue type	Intervention	Finding
Patients with cancer				
Ligibel et al.^9^	49 women with breast cancer	Breast tumor	Pre-surgical exercise	No changes in expression of Ki67, insulin receptor, and cleaved capase-3; 16 inflammation and immune gene expression pathways upregulated in exercisers vs. control
Demark-Wahnefried et al.^10^	40 men with prostate cancer	Prostate tumor	Pre-surgical caloric restriction + physical activity	Significant increase in Ki67; upregulation of genes associated with signaling pathways (GSK3B, MED12, LAMC2) and immune response (CTSL)
Patients without cancer				
Campbell et al.^8^	45 postmenopausal women	Abdominal subcutaneous adipose tissue	Caloric reduction ± exercise, or combined	No change in candidate genes by intervention group; greater weight loss was associated with a decrease in HSD17B1 and leptin expression
McTiernan et al.^11^	102 men and 100 women	Colon mucosal crypts	Exercise	Significant decrease in colon crypt cell proliferation (Ki67) in men who exercised ≥250 min/week or whose VO^2^max increased by >/=5%

Our previous Lifestyle, Exercise, and Nutrition (LEAN) trial demonstrated that a 6-month weight loss intervention was effective in producing an average 6% weight loss and 30% decrease in serum C-reactive protein levels among 100 breast cancer survivors who completed chemotherapy and/or radiation therapy^14^. Given these findings and given the paucity of research examining lifestyle interventions on tissue markers, we sought to determine the feasibility of collecting breast tissue via a baseline- and 6-month biopsy among women treated for breast cancer, and to examine the effect of the LEAN intervention on changes in body composition and breast tissue and serum biomarkers, and explore associations between 6-month changes in body composition and serum and breast tissue biomarkers.

## Results

### Recruitment and feasibility of breast biopsy

**Fig. 1:**
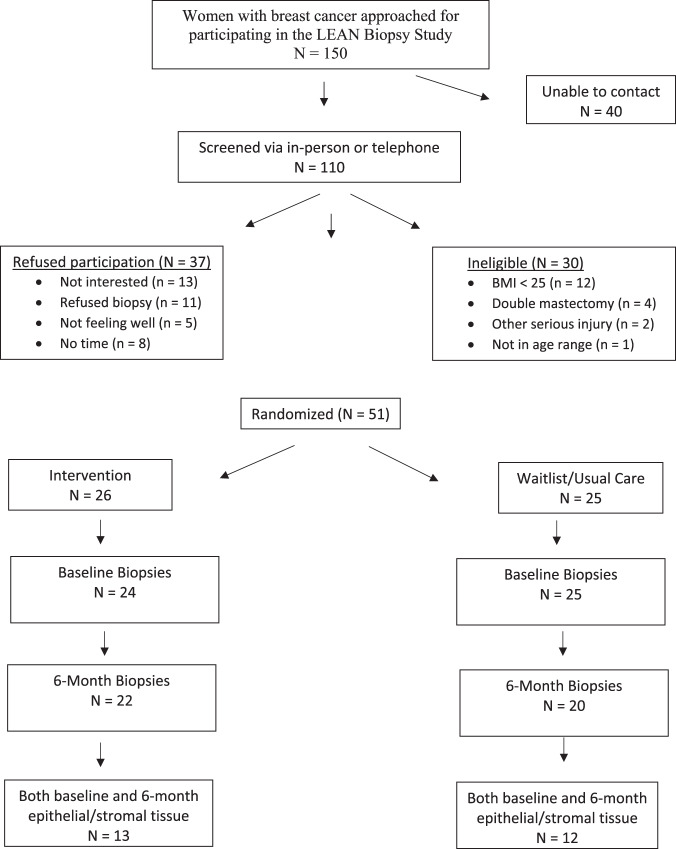
Consort diagram for the LEAN trial. Recruitment of study participants.

Between 1 February 2014 and 1 January 2016, 150 women were approached to participate in the LEAN biopsy study, with 110 women screened for participation in the trial. Fifty-one women of those screened (46%) agreed to have two breast biopsies and complete all other baseline and 6-month measures; 26 were randomized to the intervention group, and 25 were randomized to the wait-list/usual care group. Baseline and post-intervention biopsies were conducted in 49 and 42 women, respectively, with pre- and post-epithelial tissue available from 25 women (Fig. 1). Of 91 biopsies conducted, there was one adverse event related to the biopsy. At a routine oncology care clinic visit 3 weeks after the baseline biopsy, a participant reported finding a lump in her breast. After conducting a physical exam, a mammogram, and an ultrasound, the lump was determined to be a small hematoma which may have been a result of the study biopsy. The participant continued in the study, including the 6-month breast biopsy.

### Baseline characteristics

Baseline characteristics were similar for women randomized to intervention and usual care (Table 2), except for body weight and BMI which were significantly higher in the usual care group whereby the women in the usual care group more likely to be obese than the intervention group (*p* = 0.002). The mean (SD) age of participants was 56.8 (8.9) years, and their mean BMI (SD) at enrollment was 32.8 (6.0) kg/m^2^. They have diagnosed a mean of 3.3 years prior, and 54% presented with Stage I breast cancer.

**Table 2:** Baseline participant characteristics, means (SD) or *n* (%).

	Total *N* = 25	Intervention *N* = 13	Usual care *N* = 12	*P* value
Age (years)	56.8 (8.9)	59.5 (6.5)	54.1 (10.6)	0.14
Time from diagnosis (years)	3.3 (3.8)	2.8 (2.4)	3.8 (4.9)	0.51
Baseline weight (kg)	85.5 (16.2)	75.9 (7.8)	95.6 (16.8)	0.002
Baseline BMI	32.8 (6.0)	29.5 (3.6)	36.4 (6.1)	0.002
BMI ≥ 30 kg/m^2^	15 (60%)	5 (38%)	10 (83%)	0.04
Race/Ethnicity				0.10
Black or African-American	2 (8%)		2 (17%)	
Non-Hispanic White	22 (88%)	13 (100%)	9 (75%)	
Other	1 (4%)		1 (4%)	
Education				0.40
High school degree	3 (12%)	1 (8%)	2 (17%)	
Some college degree	8 (32%)	3 (23%)	5 (42%)	
College degree	6 (24%)	5 (38%)	1 (8%)	
Graduate degree	8 (32%)	4 (31%)	3 (23%)	
Postmenopausal	22 (88%)	12 (92%)	10 (83%)	0.49
Cancer stage				0.76
DCIS	4 (15%)	1 (8%)	3 (25%)	
Stage I	13 (52%)	8 (62%)	5 (42%)	
Stage II	6 (24%)	3 (23%)	3 (23%)	
Stage III	2 (8%)	1 (8%)	1 (8%)	
Current endocrine therapy				0.88
Aromatase inhibitors	10 (42%)	6 (46%)	4 (36%)	
Tamoxifen	6 (24%)	3 (23%)	3 (27%)	
None	8 (33%)	4 (31%)	4 (36%)	
Prior adjuvant treatment				0.47
None	1 (4%)	0	1 (8%)	
Radiation only	13 (52%)	8 (62%)	5 (42%)	
Chemotherapy only	1 (4%)	0	1 (8%)	
Radiation and chemotherapy	10 (38%)	5 (38%)	5 (42%)	

### Intervention adherence

Of the participants randomized to intervention, 12 of 13 women attended 100% of the 11 weight loss counseling sessions; one woman missed one session. A total of 142 weight loss counseling sessions occurred across the 13 women, with 89 (62.7%) sessions occurring in-person and 53 (37.3%) sessions occurring via telephone.

### Changes in body composition

At 6 months, there was a 6.7% weight loss in the intervention group versus a 2.0% weight gain in the usual care group (*p* < 0.0001) (Table 3). Percent body fat decreased by 4.9% in the intervention group and increased by 5.1% in the usual care group. The group difference was significant (*p* = 0.008).

**Table 3:** Baseline to 6-month change in breast tissue biomarkers, serum biomarkers, and weight compositions controlling for baseline values and baseline BMI except for BMI itself.

Variables	Intervention, mean (SD), *n* = 13	Control, mean (SD), *n* = 12	*P* value
*Tissue biomarkers*			
Baseline Ki67 (AQUA score)^a^	438.1 (225.9)	434.2 (206.2)	0.97
6-month change in Ki67	78.48 (314.6)	112.0 (403.3)	0.51
Baseline insulin receptor	3322 (2920)	2944 (1567)	0.70
6-month change in insulin receptor	893.4 (2514)	−99.7 (2329)	0.54
Baseline CD68	1237 (513.8)	1137 (362.9)	0.61
6-month change in CD68	−40.7 (471.9)	55.69 (214.5)	0.87
Baseline CD163	5277 (2416)	3882 (1830)	0.14
6-month change in CD163	−839 (3905)	1331 (3666)	0.04
*Serum biomarkers*			
Baseline Leptin, ng/ml	31.8 (14.4)	55.0 (34.0)	0.045
6-month change in Leptin	−8.6 (11.6)	3.9 (17.6)	0.03
Baseline CRP, ng/ml	3.9 (3.1)	6.8 (5.0)	0.09
6-month change in CRP	−0.8 (3.1)	−0.5 (2.0)	0.91
Baseline adiponectin, ng/ml	11.0 (4.7)	9.4 (3.9)	0.19
6-month change in adiponectin	1.8 (2.9)	−0.3 (1.2)	0.03
Baseline IGF1, ng/ml	88.30 (21.15)	101.0 (33.68)	0.27
6-month IGF1	7.01 (29.22)	0.12 (17.61)	0.52
Baseline Insulin, μU/ml	7.96 (5.41)	11.12 (5.68)	0.17
6-month change insulin	−1.47 (2.97)	−0.12 (3.53)	0.53
Baseline IL-6, pg/ml	2.61 (1.30)	4.01 (1.70)	0.03
6-month change IL-6	0.19 (2.19)	−0.29 (0.98)	0.78
Baseline TNFa, pg/ml	0.68 (0.28)	0.75 (0.30)	0.56
6-month change TNFa	−0.03 (0.32)	0.02 (0.27)	0.16
*Body composition*			
Baseline BMI (kg/m^2^)	29.54 (3.59)	36.38 (6.06)	0.002
6-month change in BMI (kg/m^2^)	−1.98 (1.31)	0.72 (0.85)	<0.0001
Baseline BMD (g/cm^−2^)	1.14 (0.09)	1.20 (0.12)	0.13
6-month change BMD (g/cm^−2^)	0.00 (0.04)	−0.01 (0.04)	0.47
Baseline lean body mass (LBM), kg	42.9 (3.1)	51.6 (7.1)	0.001
6-month change LBM, kg	−1.4 (2.5)	−1.1 (3.5)	0.57
Baseline % body fat	41.85 (3.30)	44.22 (3.72)	0.10
6-month change in % body fat	−2.03 (4.08)	2.26 (2.98)	0.008
Baseline weight, kg	75.85 (7.83)	95.64 (16.84)	0.002
6-month change in weight, kg	−5.05 (3.24)	1.88 (2.37)	<0.0001

^a^AQUA score refers to the sum of Ki-67 intensity in compartment pixel/sum of the compartment pixel area.

### Changes in serum biomarkers

Post-intervention, leptin significantly decreased by 27.0% in the intervention group compared to an increase by 7.0% in the usual care group (*p* = 0.03) (Table 3). Adiponectin significantly increased by 25.5% in the exercise group compared to a decrease by 3.2% in the usual care group (*p* = 0.03). No other biomarkers changed differently by arms (*p* > 0.05).

### Changes in breast tissue biomarkers

At 6 months, Ki67 increased 17.9% in the weight loss group and 25.8% in the usual care group (*p* = 0.51) (Table 3). Insulin receptor expression increased 26.9% in the weight loss group and decreased 3.4% in the usual care group (*p* = 0.54). CD163 significantly decreased 15.9% in the weight loss group compared to a 34.3% increase in the usual care group (*p* = 0.04). There was no significant effect of the intervention on CD68 (*p* = 0.87).

### Baseline correlation of breast tissue biomarkers with serum blood markers and body composition

At baseline, there were statistically significant inverse correlations between breast tissue levels of insulin receptor with both percent body fat (*r* = −0.47, *p* = 0.03) and serum insulin levels (*r* = −0.45, *p* = 0.04) (Table 4). There was also a significant inverse correlation between serum insulin levels and CD68 (*r* = −0.47, *p* = 0.03).

**Table 4:** Baseline correlations for body composition, serum, and breast tissue biomarkers, *n* = 25 (*r*, *p*-value).

	Ki67	IR	CD68	CD163
BMI (kg/m^2^)	0.12	−0.38	−0.09	−0.06
	0.60	0.09	0.71	0.80
BMD (g/cm^−2^)	−0.01	0.09	0.10	−0.55
	0.97	0.70	0.67	0.01
Lean body mass (kg)	−0.12	−0.23	−0.17	−0.31
	0.61	0.31	0.46	0.17
% Body fat	0.08	−0.47	0.05	−0.08
	0.72	0.03	0.83	0.74
Leptin (pg/ml)	0.17	−0.33	−0.21	−0.01
	0.48	0.14	0.34	0.95
CRP (ng/ml)	0.03	0.04	0.18	0.12
	0.88	0.87	0.43	0.60
Adiponectin (ng/ml)	0.09	−0.16	−0.22	0.30
	0.71	0.47	0.33	0.17
IGF-I (ng/ml)	0.07	0.17	0.16	0.12
	0.76	0.46	0.48	0.58
Insulin (μU/ml)	0.36	−0.45	−0.47	0.04
	0.12	0.04	0.03	0.88
IL-6 (pg/ml)	−0.13	0.11	0.03	−0.09
	0.58	0.65	0.88	0.69
TNFa (pg/ml)	−0.24	0.14	0.31	−0.26
	0.31	0.54	0.16	0.23

### Baseline to 6-month change in correlation of breast tissue biomarkers with serum blood markers and body composition

At month 6 (Table 5), change in percent body fat was inversely associated with insulin receptor (*r* = −0.42, *p* = 0.05), and change in Ki67 breast tissue expression was borderline significantly inversely associated with a change in serum levels of insulin (*r* = −0.42, *p* = 0.06). Change in CD68 breast tissue expression was inversely associated with a change in serum levels of CRP (*r* = −0.49, *p* = 0.02). Change in CD163 breast tissue expression was statistically significantly correlated with change in bone mineral density (*r* = −0.42, *p* = 0.049).

**Table 5:** Correlations for 6-month changes in body composition, serum, and breast tissue biomarkers, *n* = 25 (*r*, *p*-value).

	Ki67	IR	CD68	cd163
Leptin	−0.32	−0.13	0.120	0.09
	0.17	0.59	0.37	0.70
CRP	0.18	−0.17	−0.49	−0.08
	0.45	0.45	0.02	0.74
Adiponectin	0.05	0.14	−0.27	−0.29
	0.84	0.55	0.22	0.19
IGFI	−0.29	0.03	−0.17	−0.32
	0.22	0.90	0.44	0.14
Insulin	−0.42	0.09	−0.08	0.25
	0.06	0.70	0.71	0.27
IL6	−0.00	−0.08	−0.32	−0.16
	0.10	0.73	0.14	0.47
TNFa	−0.23	−0.17	−0.22	−0.30
	0.34	0.46	0.33	0.18
LBM	−0.19	0.09	0.21	0.11
	0.43	0.70	0.35	0.61
% Body fat	0.06	−0.42	−0.02	0.36
	0.79	0.05	0.93	0.10
BMD	0.34	0.02	−0.34	−0.42
	0.14	0.94	0.12	0.05
BMI	−0.03	−0.38	0.22	0.38
	0.90	0.09	0.33	0.08

## Discussion

A 6-month weight loss intervention in breast cancer survivors who completed chemotherapy and/or radiation therapy led to changes in body weight and fat, serum levels of leptin and adiponectin, and breast tissue levels of CD163.

Adherence to the intervention was high with 12 of 13 women attending 100% of the 11 weight loss counseling sessions. Furthermore, we demonstrated the feasibility of performing research biopsies of the breast in breast cancer survivors. Fifty-one (46%) of the women approached to participate in the study agreed to undergo two biopsies; of these 100% of participants assigned to the intervention and 92% of participants assigned to the usual care, the group underwent both biopsies. We used a core needle biopsy procedure with a 14-gauge needle without ultrasound guidance which differs from the fine needle aspirate approach used in breast cancer prevention studies^15^. Due to the biopsying of normal tissue, the use of ultrasound guidance was not required thus further ensuring cost savings and preventing scheduling challenges required for ultrasound room reservations.

To our knowledge, our study is the first to examine the effect of a weight loss intervention on breast tissue changes in women with breast cancer. The Pre-Operative Health and Body (PreHAB) study tested an aerobic and resistance training intervention consisting of supervised aerobic exercise, followed by strength training, on women diagnosed with breast cancer (*n* = 49). Additional exercise prescription consisted of home-based aerobic exercise to achieve a total of 220 min of exercise per week in a mean of 29.3 days prior to breast surgery. The study demonstrated that the intervention, which increased exercise of the patients by 200 min per week from baseline, did not change Ki67 expression but significantly reduced leptin (−12%) and upregulated cytokine–cytokine receptor interactions, NF-kB and chemokine signaling, and natural killer cell-mediated cell cytotoxicity^16^.

While both the LEAN and PreHAB studies did not report significant alterations in breast tissue following weight loss or exercise interventions, these two studies (a) demonstrated the feasibility of the collection of breast tissue and (b) laid the foundation for larger randomized controlled trials to assess lifestyle interventions on alterations in both breast tumor and healthy tissue. Other studies have examined the impact of the same in prostate tumor tissue in men with prostate cancer^10^, in abdominal subcutaneous adipose tissue in postmenopausal women without a history of cancer^8^, and in colonic mucosal crypts of men and women without a history of cancer^11^. We found that a weight loss intervention did not alter breast tissue expression of Ki67, IR, CD68; serum levels, however, were changed, with leptin and CRP decreasing, and adiponectin increasing in the presence of weight loss yet additional larger studies are warranted.

Various associations of body composition and serum and tissue biomarkers indicate the necessity of future explorations in this area. In our study, we observed body fat was inversely related to IR gene expression in the breast tissue despite a nonsignificant increase in IR gene expression in the intervention group. This suggests that with a larger sample statistical significance may have been attained. Goodwin et al. (2007) had previously reported higher expression of IR in breast tumor tissue to be independently and significantly associated with more favorable clinical outcomes including low tumor grade, lymph node negativity, progesterone positivity, and improved breast cancer and overall survival^17^. In addition, we found an inverse association between CRP and CD68/CD163. Given that CD68 and CD163 are macrophage markers of the M1 and M2 phenotype, and CRP is produced by macrophages, it is plausible that our intervention impacted the ratio of M1/M2. We had previously found that aerobic and resistance exercise intervention positively alters M1/M2 macrophage phenotype within subcutaneous abdominal adipose tissue of breast cancer survivors^18^.

It is important to recognize our examination of healthy breast tissue rather than breast tumor tissue, as women who participated in this trial had completed therapy and were free of active disease. It is known that benign breast tissue is susceptible to biomarker changes due to modifications in body weight. Fabian et al.^19^, for example, reported favorable changes in tissue levels of Ki-67 (among 15 women with measurable Ki-67), adiponectin:leptin, cyclin B1, phosphorylated retinoblastoma, and ribosomal S6 proteins in breast tissue of postmenopausal overweight or obese women who had lost weight over a 6-month period^19^. It is plausible that post-treatment weight loss interventions that alter breast tissue biology elicit a similar protective response to breast cancer recurrence.

Limitations of our study must be acknowledged. Overall, 46% of women screened for the study consented to the two biopsies, implying feasibility. Our 46% enrollment rate (51/110) is higher than we hypothesized and higher than other randomized trials of lifestyle behaviors in healthy women and women with breast cancer (e.g., 2.2% in an exercise trial of postmenopausal women^20^; 11.9% in our exercise trial in women with breast cancer^21^). The 50% (i.e., 25/51) of breast tissue yielding epithelial tissue is lower than we hypothesized yet understandable given biopsies were performed without ultrasound guidance. Using ultrasound may have increased the number of samples with epithelial tissue^22^ yet would also increase the cost of the study and also add scheduling challenges. Other study limitations are that imbalance in obesity at baseline across groups, lack of adjustment for multiple comparisons, our final sample size was underpowered for the efficacy outcomes, and in theory, biopsy specimens obtained from a single site may not be indicative of whole breast tissue alterations.

In conclusion, our weight loss trial led to favorable changes in certain metabolic and inflammatory biomarkers and body composition. This first-of-its-kind study supports the feasibility of breast tissue biopsies obtained to test the impact of lifestyle interventions on tissue biology among breast cancer survivors.

## Methods

The LEAN study (NCT02110641) was a randomized trial comparing a weight loss intervention to usual care. All measures were collected at baseline and 6 months. All procedures, including written informed consent, were approved by the Yale School of Medicine Human Investigation Committee.

### Participants and recruitment

We recruited a new cohort of women treated for breast cancer into the LEAN biopsy study. Women were recruited between 1 February 2014 and 1 January 2016 from Smilow Cancer Hospital medical oncology clinics at Yale, self-referral via study brochures in the Breast Center at Smilow Cancer Hospital, and the Yale Cancer Center Survivorship Clinic. Eligible participants were women diagnosed with breast cancer, with a BMI ≥ 25.0 kg/m^2^, who had completed chemotherapy and/or radiation therapy. Women had to be physically able to exercise, give informed consent, be accessible by telephone, and be able to communicate in English.

### Breast tissue biopsies

Core needle biopsies using a 14-gauge needle were taken from the unaffected breast following standard guidelines and protocols. Three cores were taken: one core was snap frozen, one core placed in 10% neutral buffered formalin, and one core placed in RNAlater^TM^. Both frozen tissue and tissues placed in RNAlater^TM^ solution were stored at a lab of Yale Tissue Pathology Services (YPTS) until further analysis. Formalin-fixed, paraffin-embedded biopsy specimens collected in this study were analyzed for KI67, Insulin receptor, CD68, and CD163.

### Ki67 and insulin receptor quantitative immunofluorescence (QIF) staining

Fresh cuts of LEAN study whole tissue slides were deparaffinized and rehydrated before undergoing antigen retrieval using citrate buffer (pH = 6) for 20 min at 97 °C (PT module, Lab Vision, Thermo Fisher Scientific, Waltham, MA). Slides were incubated with a solution of 0.3% hydrogen peroxide in methanol to inactivate endogenous peroxidase for 30 min, followed: 30 min of incubation with 0.3% bovine serum albumin with 0.05% Tween-20 blocking solution, incubation with a cocktail of Ki67 (mouse IgG1, Clone MIB-1, DaKo Corp, Carpinteria, CA) at 0.46 μg/ml and a wide-spectrum rabbit anti-cow cytokeratin antibody (Z0622; Dako Corp, Carpinteria, CA), followed by a 1-h incubation at room temperature with Alexa 546-conjugated goat anti-rabbit secondary antibody (A11010; Molecular Probes, Eugene, OR). Cyanine 5 (Cy5) directly conjugated to tyramide (FP1117; Perkin-Elmer, Boston, MA) at a 1:50 dilution was used as the fluorescent chromogen for Ki67 detection. Insulin receptor staining followed the same protocol with the primary antibody (Insulin receptor beta subunit monoclonal antibody, clone CT-3, mouse IgG1, Enzo Life Sciences, Farmingdale, NY) at 2 μg/ml.

### CD68 and CD163 multiplexed QIF staining

Fresh cuts of LEAN study whole tissue slides were deparaffinized and rehydrated before undergoing antigen retrieval using an EDTA buffer (pH = 8) for 20 min at 97 °C (PT module, Lab Vision, Thermo Fisher Scientific, Waltham, MA, USA). Next, we incubated the slides with a solution of 0.3% hydrogen peroxide in methanol to inactivate endogenous peroxidase for 30 min, followed by another 30 min of incubation with 0.3% bovine serum albumin with 0.05% Tween-20 blocking solution. Fluorescent staining for pancytokeratin, CD68, and CD163, biomarkers of inflammation, was performed by using a sequential multiplexed protocol with different isotype-specific primary antibodies. Antibodies against these targets were used to detect epithelial tumor cells (Anti-wide spectrum Cytokeratin antibody, Abcam, Cambridge, MA), all macrophages (CD68, mouse monoclonal IgG3, clone PG-M1, Dako Corp, 0.15 μg/mL) and M2-class macrophages (CD163, mouse monoclonal IgG1, clone 10D6 from Leica, Wetzlar, Germany, 0.006 μg/mL).

### Fluorescence signal quantification

We used the AQUA method of QIF (Navigate Biopharma), to quantify the fluorescence signal of Ki67, IR, CD68, CD163 that were measured within whole tissue compartments. QIF scores were calculated by dividing the target pixel intensity by the area of the compartment of interest^23^ and then normalized to the exposure time and bit depth at which the images were captured.

### Anthropometrics and body composition

Height was measured using a stadiometer. Weight was measured while participants were wearing light indoor clothing, without shoes. Dual-energy X-ray absorptiometry scans were performed to assess body fat, lean body mass, and bone mineral density using a Hologic 4500 scanner.

### Blood draw and serum biomarkers

A fasting (≥12 h) blood draw was performed and serum samples were stored at −80 °C until assayed. Serum concentrations of insulin, leptin, and adiponectin were measured using radioimmunoassay kits; IL-6 and TNF-α were measured using high-sensitivity enzyme-linked immunoabsorbent assay kits; and C-reactive protein (CRP) was measured using an automated chemistry analyzer.

### Physical activity and dietary intake

At baseline and 6 months, participants completed the Modifiable Physical Activity Questionnaire (MPAQ) administered by an interviewer. The past 6 months of physical activity was assessed including frequency, duration, and type using a validated physical activity questionnaire^24,25^. Past month dietary intake was assessed at baseline and 6 months by a 120-item food frequency questionnaire developed for the Women’s Health Initiative Study^26^.

### Weight loss intervention

The weight loss intervention was adapted from the Diabetes Prevention Program^27^ with updates using the 2010 US Dietary Guidelines^28^ and adapted to breast cancer survivors using the American Institute for Cancer Research/World Cancer Research Fund and American Cancer Society nutrition and physical activity guidelines^3^. The intervention included a combination of reduced caloric intake, increased physical activity, and behavioral therapy. Participants received individualized counseling sessions once per week (month 1), then every two weeks (months 2 and 3), and once per month (months, 4, 5, and 6). Each of the 11 sessions was 30 min in duration, and represented a core curriculum with specific information about nutrition, exercise, and behavior strategies from the 11-chapter LEAN book developed for the LEAN Study^14^. Given our previous study comparing in-person and telephone weight loss counseling to usual care found no difference in weight loss via in-person or telephone^12^, participants were able to choose if a session occurred in-person or via telephone. Participants recorded all food and beverage intake, minutes of physical activity and pedometer steps in the LEAN Journal on a daily basis. Participants were provided with a scale (HoMedics) to weigh themselves once per week, and recorded their weight in the LEAN journal.

Participants were instructed to reduce energy intake to the range of 1200 to 2000 kcal/day based upon baseline weight and to incur in an energy deficit of 500 kcal/day. The dietary fat goal was <25% of total energy intake. The home-based physical activity program included a goal of 150 min per week of moderate-intensity activity, such as brisk walking. Women were given a pedometer and coached to increase their number of steps to 10,000 per day. Reducing sedentary behaviors was encouraged through activities of daily living.

### Usual care group

The usual care group was provided with the American Institute for Cancer Research nutrition and physical activity brochures and referred to the Yale Cancer Center Survivorship Clinic which offers a two-session weight management program. At the completion of the study, usual care participants were offered the entire LEAN program including the 11 counseling sessions, LEAN book, and LEAN journal.

### Statistical analyses

Demographics and other baseline characteristics were summarized as means and standard deviations for continuous variables or frequencies and percentage for categorical variables. The baseline characteristics were compared using *t* test or Chi-square test as appropriate. Baseline body composition variables and biomarkers were compared using *t* test, while 6-month changes of body composition variables and biomarkers were compared between groups using the analysis of covariance (ANCOVA) method with covariate adjustment for baseline value and baseline BMI. Spearman correlation analysis was used to explore the relationship between tissue and serum biomarkers and body composition outcomes at baseline as well as 6-month change from baseline. SAS 9.4 (Cary, NC) was used for all statistical analyses. Statistical significance was set as *p* < 0.05, two-sided. Since all the analyses were exploratory, raw *p* values were reported without adjusting for multiple comparisons^29^.

### Reporting summary

Further information on research design is available in the Nature Research Reporting Summary linked to this article.

## Supplementary information

Reporting Summary

## Data Availability

The data generated or analyzed during and/or analyzed during the current study are available from the corresponding author on reasonable request.
